# Prioritizing animals for dense genotyping in order to impute missing genotypes of sparsely genotyped animals

**DOI:** 10.1186/1297-9686-46-46

**Published:** 2014-08-26

**Authors:** Xijiang Yu, John A Woolliams, Theo HE Meuwissen

**Affiliations:** Department of Animal and Aquacultural Sciences, Norwegian University of Life Sciences, PO Box 5003, Ås, 1432 Norway; The Roslin Institute and Royal (Dick) School of Veterinary Studies, University of Edinburgh, Easter Bush, Midlothian EH25 9RG UK

## Abstract

**Background:**

Genotyping accounts for a substantial part of the cost of genomic selection (GS). Using both dense and sparse SNP chips, together with imputation of missing genotypes, can reduce these costs. The aim of this study was to identify the set of candidates that are most important for dense genotyping, when they are used to impute the genotypes of sparsely genotyped animals. In a real pig pedigree, the 2500 most recently born pigs of the last generation, i.e. the target animals, were used for sparse genotyping. Their missing genotypes were imputed using either Beagle or LDMIP from *T* densely genotyped candidates chosen from the whole pedigree. A new optimization method was derived to identify the best animals for dense genotyping, which minimized the conditional genetic variance of the target animals, using either the pedigree-based relationship matrix (MCA), or a genotypic relationship matrix based on sparse marker genotypes (MCG). These, and five other methods for selecting the *T* animals were compared, using *T* = 100 or 200 animals, SNP genotypes were obtained assuming *Ne* =100 or 200, and MAF thresholds set to *D* = 0.01, 0.05 or 0.10. The performances of the methods were compared using the following criteria: call rate of true genotypes, accuracy of genotype prediction, and accuracy of genomic evaluations using the imputed genotypes.

**Results:**

For all criteria, MCA and MCG performed better than other selection methods, significantly so for all methods other than selection of sires with the largest numbers of offspring. Methods that choose animals that have the closest average relationship or contribution to the target population gave the lowest accuracy of imputation, in some cases worse than random selection, and should be avoided in practice.

**Conclusion:**

Minimization of the conditional variance of the genotypes in target animals provided an effective optimization procedure for prioritizing animals for genotyping or sequencing.

**Electronic supplementary material:**

The online version of this article (doi:10.1186/1297-9686-46-46) contains supplementary material, which is available to authorized users.

## Background

Genomic selection (GS) has been rapidly adopted by the dairy breeding industry after its introduction in 2008. Indeed, at present more than 90% of the bulls of the Holstein, Jersey, and Brown Swiss breeds in North America are genotyped every year [1]. To date, 35 populations have genomic estimated breeding values (GEBV) recognized by Interbull [2]. The success of the implementation of GS in dairy breeding has also encouraged the use of genomic evaluation in other livestock and in plants, and the exploration of its value for medical genetics. Meanwhile, it has become clear that the size of the reference population used for training is an important factor for the reliability of genomic breeding values [3, 4], which means that more genotyping is required. Therefore, there is a need for the identification of cost-effective methods to increase the size of reference populations, and reduce the cost of genotyping candidates, so that this technology becomes both more accurate and more widely applicable.

Genomic selection requires sufficiently dense SNP (single nucleotide polymorphism) chips to capture sufficient genetic variation to provide useful accuracy in predicting breeding values. However, high-density genotyping of many animals is very costly, and with the fast development of molecular technology, denser chips continue to be developed. For example, the Illumina BovineHD chip [5] that became available in 2010 has 777 962 SNPs. Another new step forward is whole-genome sequencing, e.g. the 1000 bull genome project [6]. However, the high price of whole-genome sequencing and high-density SNP chips is a barrier for the application of these new technologies to large numbers of animals, especially in species for which individual animals are not as valuable as dairy bulls.

One solution proposed by [7] was to use a mixture of dense and sparse chips together with genotype imputation, which can markedly reduce costs while maintaining high-density results. The question then arises: which animals should be densely genotyped and which sparsely? The aim of this work was to identify a set of animals in a pedigree which, when densely genotyped, provide the highest imputation accuracy. We derived a theoretical optimal strategy, both with and without genomic information, and compared it to other empirical solutions. The effectiveness of the method was assessed based on (1) the genotype imputation error rates and the correlation between true and predicted genotypes, and (2) a utilitarian measure given by the accuracy of GEBV derived with the imputed genotypes.

## Methods

### Data

A Landrace pig pedigree provided by NORSVIN AS [http://www.norsvin.no] was used. It consisted of 13 276 pigs, including 619 sires and a total of 12 generations. The 2500 most recently born animals of the last generation, all without offspring in the dataset, were chosen as the target population on which imputation would be judged. Therefore, sets of *T* pigs were selected from the entire population based on different criteria for dense genotyping (or whole-genome sequencing). Success of imputation was measured in the target population, which was sparsely genotyped. To assess the accuracy of genomic selection in the target population, 2000 animals from the target population were randomly chosen as the training set, for which phenotypes and imputed genotypes were both recorded, and the remaining 500 were treated as a validation set. GEBV were estimated and the accuracy of the GEBV was evaluated as the correlation between GEBV and true breeding values.

### Selection of animals to be densely genotyped

Seven methods were used to select sets of pigs for dense genotyping from the entire pig pedigree, including the target population. It was assumed that the budget necessary for dense genotyping of *T* individuals was available and that the target animals for testing imputation were all sparsely genotyped at density *D. T* was equal to100 or 200 and *D* was equal to 50, 100 or 200 markers per Morgan. The seven methods are described below. Two of the methods described below have optimal properties for the problem based upon minimizing the genetic variance of the target population conditional on the selected set, using either relationships obtained from Wright’s numerator relationship matrix, **A** (MCA), or a genomic relationship matrix, **G** (MCG) obtained from sparse genotyping. These criteria minimize the mean square error of an unbiased predictor of an imputed genotype from dense genotype information on the selected set. The remaining five methods are either heuristic (KIN, REL, CON, SRS) or random (RAN).

For method MCA, it is assumed that the only information available on relationships prior to dense genotyping is from the pedigree. The variance-covariance matrix for the count of a reference allele at any locus for the pedigree is proportional to Wright’s numerator relationship matrix, **A**. The variance-covariance matrix conditional on the selection of a set of animals for dense genotyping (but prior to having obtained any genotypes) is given by , where the bold subscript 1 denotes the set of target animals and bold subscript 2 denotes the set of densely genotyped animals. Thus, for example, **A**_**11**_ represents the sub-matrix of pedigree relationships among the target animals. The conditional variances, diag , are the residual variances that are expected to remain if dense genotypes were to be obtained from the selected set and used to predict the genotypes of the target set. Therefore, it is diag  that is to be minimized, and the summary statistic used was *trace*. This minimization to select the densely genotyped animals was carried out using an iterative procedure: first, the animal that most reduced *trace* was selected, i.e. animal *i* that maximized *trace* (**A**_**1***i*_**A**_*i***1**_/**A**_*ii*_), where **A**_**1***i*_ is the vector of relationships of animal *i* with the target set of animals, **A**_*i***1**_ is its transpose, and **A**_*ii*_ is the relationship of animal *i* with itself. After selecting animal *i*, the entire relationship matrix was made conditional on the genotype of animal *i*, **A**^(1)^ = **A** - **A**_: *i*_**A**_*i* :_/**A**_*ii*_ where **A**_: *i*_ is the vector of relationships of *i* with all individuals in the pedigree. **A**^(1)^ will have 0s in the row and column corresponding to the selected individual *i*. The next individual (a new *i*) was then selected to maximize *trace*, and the relationships of the entire pedigree were made conditional on this second individual to give **A**^(2)^, where . Subsequent selection then proceeded using **A**^(2)^. This iterative procedure continued until *T* animals for dense genotyping had been selected. The exact solution would require a search through all subsets of size *T*. The proposed algorithm is computationally faster and assumes that the set that is optimal for size *T* will be contained within the set that is optimal for size *T* + 1.

For method MCG, it was assumed that the sparse genotypes of the target animals were available prior to the choice of animals for dense genotyping. This allowed the numerator relationship matrix **A** to be replaced by a genomic relationship **G** matrix based upon the sparse genotyping. **G** was constructed using the FG method described by [8]: first, genotype probabilities were calculated with LDMIP [9] using only the linkage analysis options, and a relationship matrix **G** was calculated following the principles of [10], averaging over all marker positions. Candidate animals were then selected as for MCA, except that **A** was substituted by **G**. Method MCG was tested for each of the sparse marker densities (50, 100 and 200 markers per Morgan), which are denoted MCG-50, MCG-100 and MCG-200, respectively.

Four of the remaining methods are heuristic. For KIN, the *T* animals in the pedigree that have the highest mean kinship coefficients with the animals in the target population were selected. Therefore, the selected *T* animals maximize **1**^*T*^**A**_**12**_**1**, where **1** denotes an appropriately sized column vector of 1s. REL [11] differs from KIN in that it selects the *T* animals that maximize the sum of the *T-*variate regression coefficients of the allele counts for the target set on those for the selected set . (The heuristic justification for REL given in [11] of maximizing relationships between selected and target animals is actually more appropriate to KIN). For SRS, the *T* individuals that have the largest numbers of sons and daughters in the pedigree were selected. For CON, the *T* animals selected were those that had the highest genetic contribution by descent to the target population, for which contributions were obtained from **L** in the LDL^T^ decomposition of the **A** matrix, as described by [12]. Finally for RAN, a random sample of *T* animals was drawn from the whole pig pedigree without replacement.

### Simulation of genotypes and phenotypes

A forward simulator [http://ihaiwtheoserv.umb.no/tools/xform/xform.tar.gz] was used to simulate ideal populations in which SNP mutations were accumulated through generations of random mating by spontaneous mutations and recombinations. The effective sizes (*N*_*e*_) of the ideal population were 100 and 200, and the mutation rate was 10^- 8^ per base pair per meiosis. After 10 000 generations of random mating, the simulated SNP genotypes of the last generation were transmitted through the founders of the Landrace pedigree into the population by gene-dropping. The number of marker loci generated was equal to ~2200 loci/Morgan for *N*_*e*_ = 100, and ~4900 loci/Morgan for *N*_*e*_ = 200. These numbers are close to the expected results of [13] who reported 2120 loci for *N*_*e*_ = 100, and 4790 for *N*_*e*_ = 200. Among the simulated loci, proportions 0.47 and 0.52 had a minor allele frequency (MAF) less than 0.05 for *N*_*e*_ = 100 and 200, respectively. A further reduction in computing costs was obtained by simulating only one chromosome with a size of 1 Morgan (M), which will not affect the accuracy of imputation.

To test the accuracy of genomic evaluation, 30 additive QTL were randomly selected from the segregating loci, ignoring MAF. The allelic effects at the QTL followed a Laplace distribution with mean 0 and scale parameter 1. Individual phenotypes were then simulated with a heritability of 0.02 by adding NIID error terms to the individual’s breeding value, which was the sum of the allelic effects at the QTL. Although accuracy of genotype imputation is not affected by the number of chromosomes simulated and trait heritability, genomic evaluation is. The use of a low heritability offsets the small 1 M genome size, according to the concept that accuracy of genomic prediction is determined by *h*^*2*^/*Me* where *Me* is the effective number of independent segments in the genome [14].

### Genotype imputation and GEBV estimation

To simulate sparse genotyping, *D* = 50, 100, or 200 loci were randomly sampled from among all available loci, including those that were sampled for the QTL. The minimum MAF for selected marker loci was equal to 0, 0.05 or 0.10 and this was applied to the selection of loci assumed to be on the ‘sparse chip’, and to the selection of loci on the ‘dense chip’ that was used on the *T* animals used for training, and hence on genotypes to be imputed. The genotypes at the unselected loci in the sparse set were then reconstructed by using (i) Beagle [15] or (ii) LDMIP [9] which, unlike Beagle, exploits the available pedigree information. Genotypes were scored 0, 1 or 2 corresponding to the number of mutant alleles in comparison to the reference alleles. Error rates were calculated as the frequency of incorrect imputed genotypes averaged over all loci and target animals. The correlation between imputed genotypes (scored as 0, 1 and 2) and the true genotype among animals was calculated per locus and then averaged over all loci. The correlation has been shown to be much less dependent on MAF than error rates [16].

Two genomic selection methods were used for GEBV estimation, MixP [17] and GBLUP (Genomic best linear unbiased prediction) [13]. GBLUP is reported to be robust in real data analyses, but it benefits less from an increase in the marker density and is indifferent to QTL architecture. The MixP method fits a mixture of two normal distributions to the SNP effects, similar to BayesC [18], and thus attempts to give extra weight to important markers and no weight to others, while keeping computation costs to a level comparable to GBLUP. The accuracies of the GEBV from the two methods were recorded, accuracy being defined as the correlation coefficient between the true simulated breeding values and GEBV.

### Design and replication

Methods RAN, KIN, CON, SRS, and MCA were compared for all combinations of the three values of *D*, three MAF thresholds, two values of *T* and two values of *Ne.* In this complete factorial design, all methods were replicated 1000 times and imputation was done with Beagle. For MCG, because there was some variation among replicates, the computational load was much greater and therefore the comparisons for MCG were restricted to all three values of *D* with *Ne* =100, *T* = 200, and with MAF greater than 0.05. In these cases, a minimum of 400 replicates was performed, which was sufficiently large to keep standard errors small relative to effects. Comparisons of the method REL to the other methods using Beagle were restricted to 400 replicates of *T* = 100, *D* = 100, MAF > 0.05 and *Ne* = 100 only. Comparisons of all seven methods using LDMIP for imputation rather than Beagle were also restricted to 400 replicates of *T* = 100, *D* = 100, MAF ≥ 0.05 and *Ne* = 100 only.

## Results

Table 1 summarizes the number of animals shared between the lists of animals selected for dense genotyping. KIN, CON, SRS and MCA share the property that they are constant over replicates conditional on the pedigree, unlike RAN. MCG also varies over replicates due to the simulation of new genotypes, with each replicate resulting in a different **G**, and hence the selection of a different subset of *T* animals*.* Therefore, the values shown in Table 1 for MCG are the averages of the shared numbers of individuals for the different values of *D*. The strongest similarities were found between MCA and MCG. While the list obtained with SRS appeared to be the one that had the highest number of animals shared among the methods, MCA, KIN and CON have very little in common with each other. No individual was common to all four lists obtained with KIN, SRS, CON and MCA. As the sparse marker density, *D*, increased, the number of animals shared between MCG and SRS and MCA decreased.

**Table 1:** **Number of animals selected for dense genotyping shared by lists obtained using methods KIN, CON, SRS, MCA and MCG for** ***T*** **= 200 and** ***Ne*** **=100**

List	KIN	CON	SRS	MCA
**CON**	4			
**SRS**	20	20		
**MCA**	6	1	80	
**MCG-50**	9.8	5.1	93.7	121.6
**MCG-100**	9.6	4.9	89.3	111.0
**MCG-200**	8.9	4.3	82.3	100.9

Results for KIN, CON, SRS and MCA do not depend on MAF threshold and D. For MCG schemes, results vary according to the sparsity of the genotyping, *D*, and the values shown are the mean numbers shared over replicates when MAF ≥ 0.05. The mean standard error of the shared number between MCG lists and others are less than 0.15.

### Imputation accuracy

**Figure 1:**
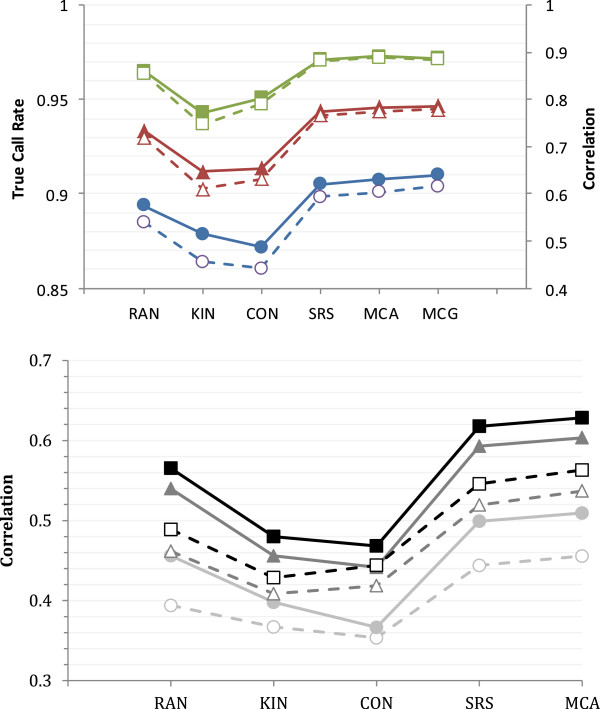
**Imputation accuracy of different methods for choosing animals for dense genotyping when using Beagle for** ***Ne*** **= 100.** Figure 1a shows the true call rate and the correlation between true and imputed genotypes for, *D* = 100, and 200 SNP/Morgan with *T* = 200 and MAF ≥ 0.05; closed symbols with solid lines and open symbols with dashed lines denote the true call rate and correlation respectively; green, red and blue denote 200, 100 and 50 SNPs/Morgan. Figure 1b shows the correlation between true and imputed genotypes for when *D* = 50 markers per Morgan for different *T* and MAF thresholds; closed symbols with solid lines and open symbols with dashed lines are *T =* 200 and 100, respectively; black, dark and light grey denote thresholds of 0.10, 0.05 and 0.00, respectively. The mean standard error of the correlation coefficients for both Figure 1a and b ranged from 2.83x10^-4^ to 1.78x10^-3^.

The imputation results are summarized in Figure 1. The proportion of markers correctly imputed and correlations between true and imputed genotypes for different values of *D* are in Figure 1a, and the correlations for different values of *T* and MAF thresholds are in Figure 1b. For all levels of *T* and *D* considered, methods MCG and MCA gave the most accurate imputation rates, although the benefits over SRS were generally not large. Imputation results using KIN or CON always gave the poorest accuracy and their performance was worse than that of RAN; typically the performance of CON was worse for *D =* 50 SNPs/M, while that of KIN was worse for higher values of *D*. Figure 1b shows the substantial benefits of increasing the size of the training set from *T* = 100 to *T* = 100 and the loss in accuracy when using and predicting alleles with low MAF. The performance of REL was similar to that of KIN and CON; for *T* = 100, *D* = 100, MAF > 0.05 and *Ne* = 100 with Beagle, the accuracy of imputation for REL was 0.56 c.f. 0.55, 0.59 and 0.70 for KIN, CON and MCA, respectively. Note that results for *T* = 200, *D* = 50, MAF = 0.05 are in both Figures 1a and b.

### GEBV accuracy with true genotypes

When *N*_*e*_ increases, the effective number of segregating segments increases [19] and the accuracy of GEBV is expected to decrease, which was observed. The genetic architecture of the design used in this study had relatively few QTL, so MixP was expected to have an advantage over GBLUP in terms of accuracy [17]. When using only loci with a MAF ≥ 0.05 instead of all loci, the difference in accuracy between MixP and GBLUP decreased; since the QTL were selected independently of their MAF, increasing the MAF threshold excluded more QTL and increased the reliance on LD with marker loci. The accuracies with true genotypes [see in Additional file 1: Table S1].

### GEBV accuracy with imputed genotypes

**Figure 2:**
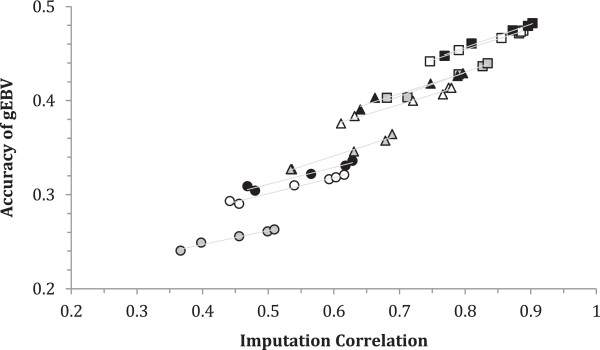
**Relationship between GEBV accuracy after imputation and the correlation between true and imputed genotypes when using GBLUP for** ***T*** **= 200 and** ***Ne*** **= 100 in the imputation training set when using Beagle.** Data are the means for each of the different methods when selecting the training set for *D* = 50, 100 and 200 SNPs/Morgan and MAF ≥ 0.00, 0.05 and 0.10; squares, triangles and circles denote 200, 100 and 50 SNPs/Morgan, respectively and symbols in black, grey and white denote MAF ≥ 0.10, 0.05 and 0.00, respectively; regression lines within MAF by *D* subclasses are also shown; identification of the different selection methods [see in Additional file 1: Table S2]; standard errors for accuracy of GEBV vary between 0.003 and 0.004.

Since changes in *N*_*e*_ did not modify the ranking of the selection methods, results for *N*_*e*_ = 200 are not shown. Figure 2 shows the GEBV accuracy against the correlation between true and imputed genotypes when using GBLUP. Accuracies using imputed genotypes should be compared to accuracies of approximately 0.50 when using GBLUP with true genotypes, and all accuracies were less than this value, as expected. The differences between the selection methods mirrored those for imputation accuracy although differences in GEBV accuracy were smaller. [see Additional file 1: Table S2] contains the data that was used to generate Figure 2 and that permit the selection methods to be distinguished. Methods MCG and MCA gave the most accurate GEBV, while the accuracy was slightly lower with SRS. Typically, KIN gave the least accurate GEBV. The average results of the other sets lie in between these extremes. The effects of the different strategies for selection on the GEBV accuracy following imputation from MixP were similar to those for GBLUP, although the magnitudes of the accuracies were slightly greater when using MixP; these are not shown in Figure 2. It is clear that both between and within combinations of MAF and *D*, the accuracy of the GEBV increased with the correlation between the true and imputed genotypes. Figure 2 also shows the regression of GEBV accuracy with GBLUP on genotype imputation accuracy within each MAF and *D* subclass.

Clearly, the accuracy of GEBV increases with imputation accuracy. One interesting phenomenon is that higher genotype imputation accuracy does not necessarily mean higher GEBV accuracy (Figure 1). For example, with a sparse marker density of 50 SNPs/Morgan, the imputation result is better with KIN than with CON, but the opposite occurred for the accuracy of GEBV estimations. This might be because KIN yielded greater imputation accuracy so close relatives were very accurately imputed, but the final GEBV had poorer accuracy because the less related animals impacted GEBV estimation. This phenomenon is observed in Figure 2 across values of *D,* where equal accuracy of imputed SNPs does not directly translate to equal accuracy of GEBV; for example, this is the case when comparing 100 and 200 SNPs/Morgan for imputation accuracy of ~ 0.8, perhaps because the denser SNP chip has more known genotypes.

The effect of imputation accuracy on GEBV accuracy was studied in more detail by fitting linear models to the data on the accuracy of imputation and accuracy of GEBV when using GBLUP. Averaging over the selection methods for each *T* × *D* × MAF subclass, the regression coefficient of the mean GEBV accuracy on mean imputation accuracy for the 18 subclasses was 0.491 (standard (se) = 0.008), i.e. increasing imputation accuracy by 0.01 gave 0.005 extra accuracy in GEBV. However, within these subclasses, the pooled regression coefficient of GEBV accuracy on imputation accuracy was lower, i.e. 0.218 (se = 0.009), but with evidence (P < 0.05) of variation in this regression among the 18 subclasses. Conditional on the observed imputation accuracy, the size of the imputation training set *T* had no further effect on the accuracy of GEBV, which might be as expected, and there was only a small trend (P < 0.1) for the MAF threshold to have additional effects on accuracy, other than through imputation accuracy. However, marker density *D* had a strong effect on accuracy of GEBV after accounting for its effect on imputation (P < 0.001); compared to 50 SNPs/Morgan, accuracy of GEBV increased by an additional 0.031 (se = 0.13) and 0.070 (se = 0.013) units for *D* = 100 and 200 SNP/Morgan in this dataset, with an average accuracy of 0.4.

**Figure 3:**
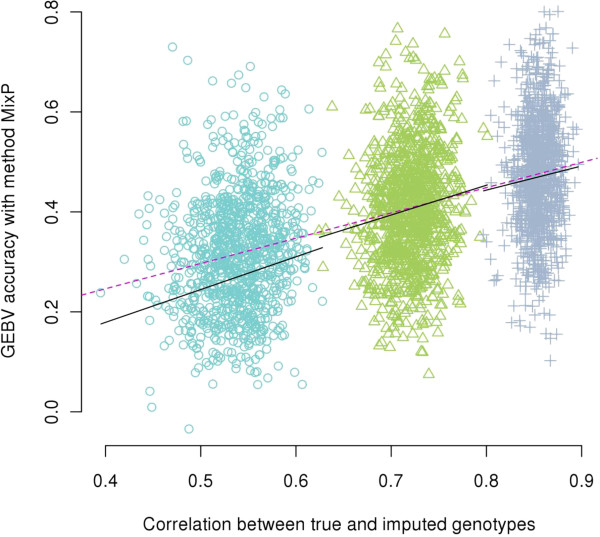
**Regression of the GEBV accuracy on the correlation between true and imputed genotypes for method RAN with varying** ***D*** **when using Beagle.** The three groups of points correspond to, from left to right, 50, 100, and 200 markers per Morgan; dashed line is the regression line for all the data points and solid lines are the local regression lines fitted within each level of *D*.

Figure 3 shows all the data on accuracies of imputation and GEBV obtained with RAN. As *D* increased, the variance of imputation accuracy among replicates decreased faster than the variance of the accuracy of GEBV, and the regression of GEBV accuracy on imputation accuracy decreased, which suggests diminishing return in GEBV accuracy from increasing imputation accuracy. The variance of GEBV accuracy was larger than the variance of imputation accuracy because GEBV estimation has more sources of error.

### Comparison of Beagle and LDMIP

Qualitatively, the results obtained when using LDMIP for imputation were largely similar to those when using Beagle. In particular, SRS, MCA and MCG remained superior to KIN, CON and REL in imputation accuracy and GEBV accuracy when using LDMIP instead of Beagle. LDMIP increased imputation accuracy compared to Beagle, with the correlation for MCA increasing from 0.70 (se = 0.001) to 0.80 (se = 0.001) for *T* = 100, *D* = 100, MAF > 0.05 and *Ne* = 100 [see in Additional file 1: Table S1]. With RAN, Beagle and LDMIP gave very similar accuracies, but for the lists that depend on relationships, LDMIP achieved greater imputation accuracy; this might be as expected since LDMIP uses family information for the imputation. The benefits of LDMIP in terms of GEBV accuracy were smaller in magnitude than for imputation accuracy. For example the accuracy of GEBV for MCA increased from 0.384 to 0.408 (se of the difference = 0.006).

## Discussion

We have compared seven methods for prioritizing animals for dense genotyping, with the objective of imputing a set of target animals up to high-density genotype information. Although there was no selection to create the data used for testing, the commercial pedigree used was developed by selection. The two novel methods, MCA and MCG, that were developed for this objective, were shown to be superior over other methods, including heuristic methods based on contributions (CON, SRS) and relationships (KIN, REL). Somewhat surprisingly, there was relatively little overlap in animals between the lists, although the lists for SRS, MCA and MCG had considerable overlap. These last two lists, closely followed by SRS, were consistent in achieving the highest imputation accuracies. The list obtained with CON had a lower imputation accuracy because it tended to choose older ancestors that had been favored over generations by selection, hence making large contributions to the target population. However, since these ancestors are often only distantly related (through generations) to the target animals, the haplotypes they have in common are rather short and thus more difficult to detect by the imputation software. KIN will tend to focus on relationships with subsets of the target population and not with the whole population, whereas MCA and MCG use relationships but make the choice conditional on the impact of the set as a whole. The previously published method, REL, was markedly inferior [11] and its performance was similar to that of KIN and CON. The differences in overall performance of all methods in [11] were similar to those of REL (see the large difference in overall performance between MCA and REL observed here). One of the modifications (IDTS) carried out in [11] adapts REL, but it makes the selection criterion for dense genotyping independent of the impact of those selected on the target population. Other criteria in [11] consider heuristic empirical scoring criteria and so are unlikely to be easily generalized, even if performing well in particular situations.

Minimizing the conditional variance based on the **A** matrix (MCA**)** makes sense since the aim is to predict the missing genotypes and thus, conditional on (dense) genotypic information, to minimize the unexplained genetic variation. In the absence of any further information, we used the pedigree relationship matrix **A** to describe the genetic (co)variances between the animals. In the presence of sparse genotype information, we used the genomic relationship matrix **G** for this, i.e. the MCG method. One can also imagine a situation where some animals are sparsely genotyped and some are not genotyped, in which case the one-step method could be used to set up a relationship matrix across these two types of animals, **H** [8, 20, 21]. Similar to MCG, the **H** matrix could be used to minimize the conditional variances i.e. the MCH method. In this case, it will be important to regress the sparse-markers-based **G** matrix back to the **A** matrix [19] (see [8] for incorporation into the one-step method) in order to account for the imperfect prediction accuracy of sparse markers.

The observation that sires with the largest number of sons and daughters gave good GEBV accuracy is reasonable since (1) in our example, they contributed most genomes to the target animals; and (2) they are a set of relatively distantly related animals that are in part selected in order to avoid inbreeding in the breeding scheme. Experience from applying this methodology to other commercial pedigrees (not shown here) supports large but imperfect correspondence between the lists arising from MCA and the individuals with the largest number of offspring in the target population. Interestingly, random lists performed reasonably well and resulted in more accurate GEBV than CON and KIN, and may be considered because of its ease of implementation.

The empirical positive relationship between imputation accuracy and accuracy of GEBV when using the imputed genotypes needs to be interpreted with care since it is not absolute. For example, imputation accuracy was increased by avoiding the imputation (prediction) of genotypes at loci with low MAF (see Figure 1b). However, when true genotypes were used, the inclusion of low MAF loci increased the accuracy of the GEBV [see in Additional file 1: Table S1], contrary to this empirical relationship, although the ranking of the methods for selecting those for dense genotyping was not affected. Thus, in a genomic selection scheme, loci with low MAF should not be ignored.

## Conclusions

We proposed and tested two novel criteria (MCA and MCG) for prioritizing animals for dense genotyping when the intended use of the dense genotyping is to impute the missing marker data on sparsely genotyped animals in a target population. The two criteria apply to (i) when only pedigree information is available, MCA, and (ii) when all animals are already sparsely genotyped, MCG. MCA and MCG minimize the conditional genetic variance in the target population based on, respectively, the numerator relationship matrix **A** and the genomic **G** matrix calculated from the sparse genotypes. The simulation study showed that the new criteria resulted in higher imputation accuracies of the missing genotypes than alternative criteria such as selecting a random set of animals for dense genotyping, selecting sires with the largest number of offspring, selecting animals that were most related to the target population, or selecting animals that had the highest genetic contributions to the target population.

## Electronic supplementary material

Additional file 1: Table S1: Provides the accuracies of GEBV when all genotypes for all animals are known without error. **Table S2.** Provides the accuracies of GEBV when using imputed genotypes for six different methods of selecting animals for high-density genotyping, three MAF thresholds for inclusion of loci in the estimation process and three densities (*D)* of sparse genotyping for imputation (SNP/Morgan). Genotypes were generated assuming *N*_*e*_ = 100; standard errors for all values vary between 0.003 and 0.005. **Table S3.** Compares imputation performances when using either Beagle or LDMIP for six methods of selecting animals for high-density SNP information. The measures compared are imputation rate (fraction of correctly imputed genotypes); imputation accuracy (correlation of true and imputed genotype); and accuracies of genomic evaluations when using GBLUP or Mix-P. Results are given for *T* = 100, *D* = 100, MAF ≥ 0.05 and *Ne* = 100. (DOCX 21 KB)
