# Supplementary material for: Prioritizing animals for dense genotyping in order to impute missing genotypes of sparsely genotyped animals
Source: Genet Sel Evol. 2014 Aug 26;46(1):46. doi: 10.1186/1297-9686-46-46 (PMC4283150; doi:10.1186/1297-9686-46-46)
Supplement: Supplementary file 1 — Additional file 1: Table S1: Provides the accuracies of GEBV when all genotypes for all animals are known without error. Table S2. Provides the accuracies of GEBV when using imputed genotypes for six different methods of selecting animals for high-density genotyping, three MAF thresholds for inclusion of loci in the estimation process and three densities (D) of sparse genotyping for imputation (SNP/Morgan). Genotypes were generated assuming N e = 100; standard errors for all values vary between 0.003 and 0.005. Table S3. Compares imputation performances when using either Beagle or LDMIP for six methods of selecting animals for high-density SNP information. The measures compared are imputation rate (fraction of correctly imputed genotypes); imputation accuracy (correlation of true and imputed genotype); and accuracies of genomic evaluations when using GBLUP or Mix-P. Results are given for T = 100, D = 100, MAF ≥ 0.05 and Ne = 100. (DOCX 21 KB) [file 12711_2013_2628_MOESM1_ESM.docx]

**Additional file 1: Tables S1, S2 and S3**

**Additional file 1: Table S1**

The accuracies of GEBVs when all genotypes for all animals are known without error. This is shown for two methods of evaluation (MixP and GBLUP), for two thresholds for including loci in the evaluations (MAF ≥ 0 and 0.05), and for two values of *Ne* for generating genotypes (*Ne* = 100 and 200). Values shown are means of 200 replicates.

| ***Ne*** | **MAF threshold** | **MixP** | **GBLUP** |
| --- | --- | --- | --- |
| 100 | 0 | 0.61 | 0.52 |
|  | 0.05 | 0.54 | 0.48 |
| 200 | 0 | 0.64 | 0.53 |
|  | 0.05 | 0.58 | 0.50 |

**Additional file 1: Table S2**

The accuracy of GEBV estimation when using GBLUP for 6 different methods of selecting *T* = 200 animals for high density genotyping, 3 MAF thresholds for including loci in the estimation process and 3 densities (*D)* of sparse genotyping for imputation (SNP/Morgan). Genotypes were generated assuming *N_e_* =100. Standard errors for all values vary between 0.003 and 0.005.

| **MAF** | ***D*** | **RAN** | **KIN** | **CON** | **SRS** | **MCA** | **MCG** |
| --- | --- | --- | --- | --- | --- | --- | --- |
| 0 | 50 | 0.256 | 0.249 | 0.240 | 0.261 | 0.263 | - |
| 0 | 100 | 0.346 | 0.327 | 0.327 | 0.357 | 0.364 | - |
| 0 | 200 | 0.428 | 0.403 | 0.403 | 0.437 | 0.440 | - |
|  |  |  |  |  |  |  |  |
| 0.05 | 50 | 0.310 | 0.290 | 0.293 | 0.316 | 0.318 | 0.321 |
| 0.05 | 100 | 0.400 | 0.376 | 0.383 | 0.407 | 0.414 | 0.413 |
| 0.05 | 200 | 0.466 | 0.442 | 0.454 | 0.471 | 0.474 | 0.473 |
|  |  |  |  |  |  |  |  |
| 0.1 | 50 | 0.322 | 0.304 | 0.309 | 0.331 | 0.336 | - |
| 0.1 | 100 | 0.418 | 0.391 | 0.403 | 0.426 | 0.429 | - |
| 0.1 | 200 | 0.475 | 0.447 | 0.461 | 0.479 | 0.482 | - |

**Additional file 1: Table S3**

Comparison of imputation performance when using either Beagle or LDMIP for six methods of selecting animals for high density SNP information. The measures compared are imputation rate (fraction of correctly imputed genotypes); imputation accuracy (correlation of true and imputed genotype); and accuracies of genomic evaluations when using GBLUP or Mix-P. Results are for *T*=100, *D*=100, MAF ≥ 0.05 and *Ne* =100.

|  | **RAN** | **KIN** | **REL** | **CON** | **SRS** | **MCA** |
| --- | --- | --- | --- | --- | --- | --- |
| *Imputation Rate* |  |  |  |  |  |  |
| Beagle | 0.92 | 0.90 | 0.90 | 0.91 | 0.93 | 0.93 |
| LDMIP | 0.92 | 0.92 | 0.92 | 0.93 | 0.95 | 0.95 |
| *Imputation Accuracy* |  |  |  |  |  |  |
| Beagle | 0.64 | 0.55 | 0.56 | 0.59 | 0.69 | 0.71 |
| LDMIP | 0.63 | 0.62 | 0.64 | 0.70 | 0.80 | 0.80 |
| *GBLUP Accuracy* |  |  |  |  |  |  |
| Beagle | 0.37 | 0.35 | 0.40 | 0.36 | 0.38 | 0.38 |
| LDMIP | 0.37 | 0.36 | 0.40 | 0.40 | 0.41 | 0.41 |
| *Mix-P Accuracy* |  |  |  |  |  |  |
| Beagle | 0.38 | 0.36 | 0.39 | 0.37 | 0.39 | 0.39 |
| LDMIP | 0.38 | 0.38 | 0.38 | 0.41 | 0.43 | 0.42 |
